# Supplementary figures and images for: Enhancement-constrained acceleration: A robust reconstruction framework in breast DCE-MRI
Source: PLoS One. 2021 Oct 28;16(10):e0258621. doi: 10.1371/journal.pone.0258621 (PMC8553053; doi:10.1371/journal.pone.0258621)

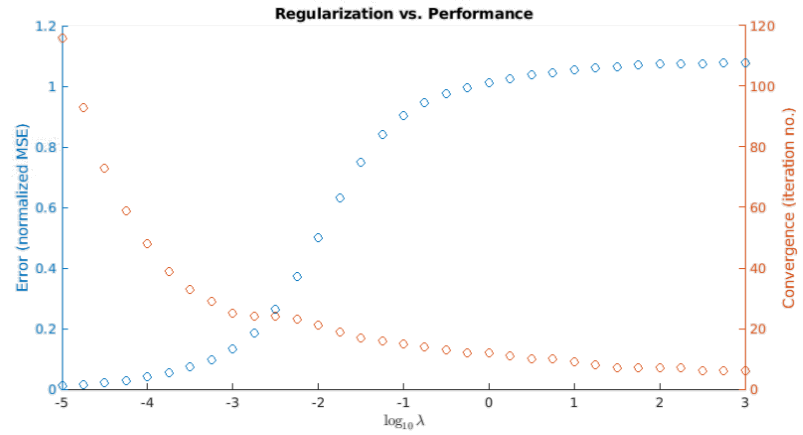

Supplement: S1 Fig — (Blue) Error, measured here by normalized mean-square error (MSE), increases with regularization strength. (Red) Computation time, measured in number of iterations, decreases with regularization strength. (TIF) [file pone.0258621.s001.tif]
