# Supplementary material for: Enhancement-constrained acceleration: A robust reconstruction framework in breast DCE-MRI
Source: PLoS One. 2021 Oct 28;16(10):e0258621. doi: 10.1371/journal.pone.0258621 (PMC8553053; doi:10.1371/journal.pone.0258621)
Supplement: S1 Appendix — (DOCX) [file pone.0258621.s002.docx]

**S1 Appendix: Formal Description of Partition Constraints**

Suppose a total of N measurements are taken over the course of the scan: denote these as $Y=\left( y_{1},\ldots,y_{N} \right)$. Choose a reconstruction temporal resolution such that we have $T$ time points in the reconstructed image. We may identify each measurement with the time $t_{n}\in\{1,\ldots,T\}$ at which it was taken. For example, if we partition the time duration of the scan into $T$ intervals, then $t_{n}=1$ indicates the $n$th measurement was acquired during the first time interval. We will also write $v_{n}\in\{1,\ldots,V\}$ to denote the$k$-space voxel (i.e., spatial frequency) at which measurement $n$ was acquired. Our method does not require that we observe an equal number of measurements during each time interval $t=1,\ldots,T$, but we typically expect to have $N/T$ measurements for each $t$.

Let $X=\left( X_{1},\ldots,X_{T} \right)$ be a dynamic timeseries image with $T$ time-points, where each static image $X_{t}$ has $V$ voxels; this is the unknown sequence of true images (up to spatiotemporal discretization) that we aim to reconstruct. In a fully sampled regime ($N=VT$), we would observe the complete $k$-space data $\tilde{X}_{t}=\mathcal{F}X_{t}$ at each time $t=1,\ldots,T$, where $\mathcal{F}$ is the $V\times V$ discrete Fourier transform matrix. In other words, we would measure (a noisy version of) the sequence

$$\tilde{X}=\left( \tilde{X}_{1},\ldots,\tilde{X}_{T} \right)=\left( \mathcal{F}X_{1}\mathcal{,\ldots,F}X_{T} \right)=\left( I_{T}\otimes\mathcal{F} \right)X$$

where $\otimes$ denotes the Kronecker product. (Abusing notation, we will interpret $X$ and $\tilde{X}$ as either $V\times T$ matrices or vectors of length $VT$, depending on the context.) When reconstructing at accelerated time resolution ($N<VT$), the measured data is a proper subset of the full timeseries. Define

$$\Omega=\left( \left( t_{1},v_{1} \right),\ldots,\left( t_{N},v_{N} \right) \right)$$

as the sequence of ***(time-index, spatial frequency-index)*** pairs at which the $k$-space measurements $y_{1},\ldots,y_{N}$ were taken (i.e., each entry in $\Omega$ lies in the set $\left\{ 1,\ldots,T \right\}\times\left\{ 1,\ldots,V \right\}$). Then $\tilde{X}_{\Omega}$ is the observed part of the Fourier transform of the dynamic image sequence. For each $n$, our observation $y_{n}$ is the voxel $v_{n}$ from the image $\tilde{X}_{t_{n}}$, giving the relation $y_{n}=\left( \tilde{X}_{t_{n}} \right)_{v_{n}}+noise$. The remaining entries in $\tilde{X}$ (i.e., those in $\tilde{X}_{\Omega^{c}}$) are left unobserved and must be reconstructed by our algorithm. Our data fidelity constraint stipulates that the observed $k$-space data $Y$ must remain unaltered by the reconstruction. Therefore, any reconstruction $\hat{X}$ of $X$ must satisfy

$$\begin{aligned} \left[ \left( I_{T}\mathcal{\otimes F} \right)\hat{X} \right]_{\left( \Omega\right)}=Y_{\Omega}. \end{aligned}\left( 1 \right)$$
