# Supplementary material for: Enhancement-constrained acceleration: A robust reconstruction framework in breast DCE-MRI
Source: PLoS One. 2021 Oct 28;16(10):e0258621. doi: 10.1371/journal.pone.0258621 (PMC8553053; doi:10.1371/journal.pone.0258621)
Supplement: S2 Appendix — (DOCX) [file pone.0258621.s003.docx]

**S2** **Appendix: The Penalty Function**

After requiring that each reconstructed image match the $k$-space points in the partition corresponding to its interval, we minimize over a weighted and regularized smoothness penalty to determine a unique reconstruction. The mathematical details of this process are laid out in this section.

First, we will define the loss function minimized by our reconstruction optimization: the discretized curvature. For a given signal $x=\left( x_{1},\ldots,x_{T} \right)$ composed of $T$ time points, define the smoothness penalty function $S$ as the $l^{2}$-norm of its discrete second derivative:

$$S\left( x \right)=\sum_{t=2}^{T-1} \left| \left( x_{t+1}-x_{t} \right)-\left( x_{t}-x_{t-1} \right) \right|^{2}.$$

Since $S$ is a quadratic function of $x$, we may write it in terms of a linear operator $D$ satisfying

$$S\left( x \right)=x^{*}Dx.$$

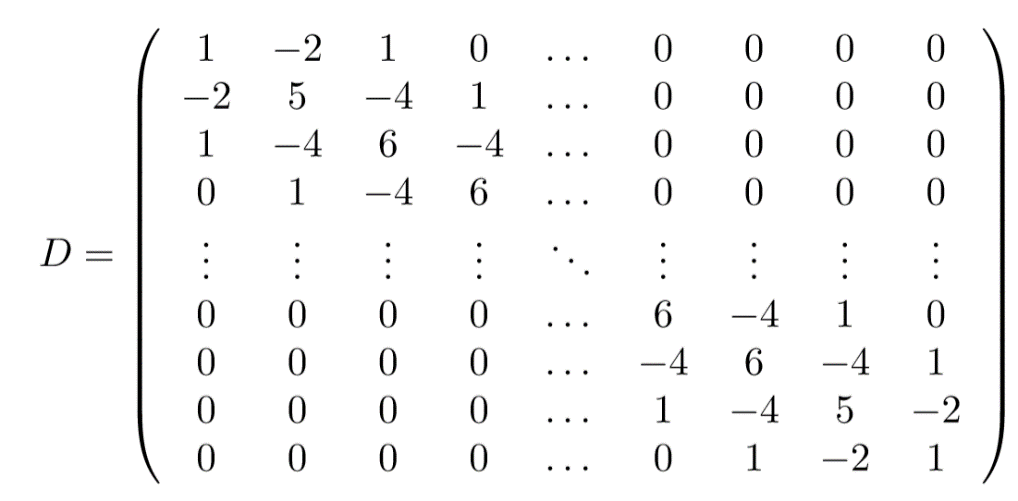
As a matrix, $D$ takes the form

The operator $D$ is poorly conditioned. In fact, it can be shown that the respective supremum and infimum of eigenvalues of $D$ (over all values of $T$) are $\sigma_{max}\left( D \right)=16$ and $\sigma_{min}\left( D \right)=0$.

Since $D$ is (nearly) degenerate and we will later need to invert it, we add a small parameter $\lambda$ (we chose $\lambda={10}^{-5}$) to regularize the operator $D$:

$$D_{\lambda}=D+\lambda I_{T},$$

where $I_{T}$ is the $T$-dimensional identity. Thus, in the reconstruction process, we use the regularized smoothness penalty

$$S_{\lambda}\left( x \right)=x^{*}D_{\lambda}x.$$

If $X=\left( X_{1},\ldots,X_{T} \right)$ is a dynamic image composed of $T$ static images, each with $V$ voxels, then we can extend $S_{\lambda}$ to act on $X$ by

$$S_{\lambda}\left( X \right)=\sum_{v=1}^{V} x_{v}^{*}D_{\lambda}x_{v},$$

where $x_{v}$ is the timeseries signal at the $v^{\text{th}}$ voxel.

It may also be desirable to penalize a lack of smoothness in some voxels more than in others. To enforce such a prioritization of “interesting” voxels, we may also include voxel-wise weighting terms $w_{v}$, generating our full loss function $L$:

$$L\left( X \right)=\sum_{v=1}^{V} w_{v}x_{v}^{*}D_{\lambda}x_{v}.$$

Setting $W$ as the $V\times V$ diagonal matrix with entries $w_{v}$, we can write the reconstruction optimization problem as

$$\begin{aligned} \hat{X}=\underset{X}{argmin} \left\{ \left\langle W,XD_{\lambda}X^{*} \right\rangle\mid\left[ \left( I_{T}\mathcal{\otimes F} \right)\hat{X} \right]_{\left( \Omega\right)}=Y_{\Omega} \right\}.\#\left( 2 \right) \end{aligned}$$
