# Supplementary material for: Enhancement-constrained acceleration: A robust reconstruction framework in breast DCE-MRI
Source: PLoS One. 2021 Oct 28;16(10):e0258621. doi: 10.1371/journal.pone.0258621 (PMC8553053; doi:10.1371/journal.pone.0258621)
Supplement: S3 Appendix — (DOCX) [file pone.0258621.s004.docx]

**S3 Appendix: Solutions to the Optimization Problem**

*General Case: Arbitrary Voxel Weights*

In the following section, we will show that the optimization problem posed by the reconstruction has the following unique solution:

$$\begin{aligned} \hat{X}=\left[ D_{\lambda}^{-1}\otimes W^{-1}\mathcal{F}^{*} \right]_{*,\Omega}\cdot\left( \left[ D_{\lambda}^{-1}\otimes\mathcal{F}W^{-1}\mathcal{F}^{*} \right]_{\Omega,\Omega} \right)^{-1}\cdot Y, \\ \left( 3 \right) \end{aligned}$$

where, for a $VT\times VT$ matrix $M$, $M_{,\Omega}$ denotes the $VT\times\vee\Omega\vee$ submatrix of $M$ with columns belonging to $\Omega$; similarly, $M_{\Omega,\Omega}$ is the $\Omega\vee\times\vee\Omega\vee$ submatrix of $M$ consisting of rows and columns in $\Omega$.

Since computing this solution for $\hat{X}$ requires a large matrix inversion $\left( N\times N\sim{10}^{16} \right)$, we implement this computation iteratively. We initialize the solution in $k$-space, estimating the Fourier-transformed image sequence $\tilde{X}$ by zero-filling around the $N$ $k$-space measurements of $Y$ ($\tilde{X}_{\Omega}=Y$ and $\tilde{X}_{\Omega^{c}}=0$). We then descend along the conjugate gradient of the smoothness penalty until we converge to $\left( 3 \right)$. See code for implementation: https://github.com/tyo8/ECA_Demo.

We will now check our solution to the target optimization problem. First, we will show that $\left( 3 \right)$ satisfies the problem constraints.

$$\left[ \left( I_{T}\mathcal{\otimes F} \right)\hat{X} \right]_{\left( \Omega\right)}= \left[ \left( I_{T}\mathcal{\otimes F} \right)\left[ D_{\lambda}^{-1}\otimes W^{-1}\mathcal{F}^{*} \right]_{*,\iota\left( \Omega\right)}\cdot\left( \left[ D_{\lambda}^{-1}\otimes\mathcal{F}W^{-1}\mathcal{F}^{*} \right]_{\iota\left( \Omega\right),\iota\left( \Omega\right)} \right)^{-1}\cdot Y_{\Omega} \right]_{\left( \Omega\right)}$$

$$= \left[ D_{\lambda}^{-1}\otimes\mathcal{F}W^{-1}\mathcal{F}^{*} \right]_{*,\left( \Omega\right)}\cdot\left( \left[ D_{\lambda}^{-1}\otimes\mathcal{F}W^{-1}\mathcal{F}^{*} \right]_{\left( \Omega\right),\left( \Omega\right)} \right)^{-1}\cdot Y_{\Omega}$$

Thus, $\hat{X}$ is a feasible solution to the optimization problem.

Next, we will show that $\left( 3 \right)$ satisfies first-order optimality conditions. Since $D_{\lambda}$ and $W$ are both positive-definite matrices, an optimal solution is unique. To see that $\hat{X}$ is optimal, we must show that the gradient of the loss function lies in the span of the gradient of the constraints.

$$\nabla_{X}\mathcal{L}\left( \hat{X} \right)=\left( D_{\lambda}\otimes W \right) \hat{X}$$

$$= \left( D_{\lambda}\otimes W \right)\cdot\left[ D_{\lambda}^{-1}\otimes W^{-1}\mathcal{F}^{*} \right]_{*,\left( \Omega\right)}\cdot\left( \left[ D_{\lambda}^{-1}\otimes\mathcal{F}W^{-1}\mathcal{F}^{*} \right]_{\left( \Omega\right),\left( \Omega\right)} \right)^{-1}\cdot Y_{\Omega}$$

$$=\left[ \left( D_{\lambda}\otimes W \right)\cdot\left( D_{\lambda}^{-1}\otimes W^{-1}\mathcal{F}^{*} \right) \right]_{*,\left( \Omega\right)}\cdot\left( \left[ D_{\lambda}^{-1}\otimes\mathcal{F}W^{-1}\mathcal{F}^{*} \right]_{\left( \Omega\right),\left( \Omega\right)} \right)^{-1}\cdot Y_{\Omega}$$

$$=\left[ I_{T}\otimes\mathcal{F}^{*} \right]_{*,\left( \Omega\right)}\cdot\left( \left[ D_{\lambda}^{-1}\otimes\mathcal{F}W^{-1}\mathcal{F}^{*} \right]_{\left( \Omega\right),\left( \Omega\right)} \right)^{-1}\cdot Y_{\Omega}$$

Since $\left[ I_{T}\otimes\mathcal{F}^{*} \right]_{*,\left( \Omega\right)}$ is the gradient of the constraint function, we’ve shown that $\nabla_{X}\mathcal{L}\left( \hat{X} \right)$ lies in the span of the constraint gradients. It follows that $\hat{X}$ is the unique solution to the reconstruction optimization problem.

*Special Case: Uniform Voxel Weights*

When all voxels are uniformly weighted ($W\propto I_{V}$), the solution simplifies significantly:

$$\hat{\hat{X}}=\left( I_{T}\otimes\mathcal{F}^{*} \right)\overline{Y},$$

where $\bar{Y}=\left[ D_{\lambda}^{-1}\otimes I_{V} \right]_{*,\Omega}\cdot\left( \left[ D_{\lambda}^{-1}\otimes I_{V} \right]_{\Omega,\Omega} \right)^{-1}\cdot Y_{\Omega}$.
