# Supplementary material for: Enhancement-constrained acceleration: A robust reconstruction framework in breast DCE-MRI
Source: PLoS One. 2021 Oct 28;16(10):e0258621. doi: 10.1371/journal.pone.0258621 (PMC8553053; doi:10.1371/journal.pone.0258621)
Supplement: S4 Appendix — (DOCX) [file pone.0258621.s005.docx]

S4 **Appendix: Regularization in the Optimization Problem**

Recall the penalty function defined above:

$$S\left( x \right)=x^{*}Dx.$$

Since the operator $D$ is not invertible (and must be inverted to efficiently compute the optimal solution), we add a small diagonal element $\lambda I_{T}$ to $D$ to get the regularized penalty function

$$S_{\lambda}\left( x \right)=x^{*}D_{\lambda}x.$$

As with any regularization parameter, different values of $\lambda$ offer different trade-offs between the optimality of the solution and the speed at which it is reached. We hope to choose $\lambda$ large enough that $D_{\lambda}$ is well-conditioned and small enough that $S_{\lambda}-S$ is small compared to $S$. Equivalently, we require that

$$\lambda^{-1}\gg\frac{\langle W,XX^{*}\rangle}{\langle W,XDX^{*}\rangle}$$

and $-{log}_{10}\lambda\leq k$ for some integer $k$ (which should be chosen empirically based on problem size, system requirements, and the conditioning of the weights matrix $W$). We chose $\lambda={10}^{-5}$ because it converged sufficiently quickly and did not significantly alter the penalty function computed in our experiment. To illustrate the regularization-vs-performance tradeoff, a pair of plots for different values of $\lambda$ in a small-scale ($VT\sim{10}^{6}$) reconstruction are shown below (**Fig S1**). As regularization increases, we converge more quickly to a solution, but that solution is less accurate.
