# Supplementary material for: Enhancement-constrained acceleration: A robust reconstruction framework in breast DCE-MRI
Source: PLoS One. 2021 Oct 28;16(10):e0258621. doi: 10.1371/journal.pone.0258621 (PMC8553053; doi:10.1371/journal.pone.0258621)
Supplement: Attachment — Submitted filename: PLOS_DCE_review.docx [file pone.0258621.s006.docx]

Comments to the Author

The work demonstrates a new reconstruction method for semi-qualitative breast DCE-MRI data. In particular, the authors claim that the method provides high fidelity reconstruction at 0.25s temporal resolution. The work develops a virtual scanner which is used for simulating k-space data and subsequent sampling. This is a useful contribution. Also, the code has been shared on Github useful for reproducible research. However, I have some concerns with the data simulations, assumptions and claims (see minor comments related to opinions) which are detailed below.

Major Comments: 

1. In silico phantom: The standard phantom for quantitiative breast DCE is the QIBA phantom (<https://qibawiki.rsna.org/index.php/Main_Page>) that provides specific qMRI pharmacokinetic parameters against which image reconstruction, image processing, regression analysis methods are typically compared. Although the idea of starting with “clinical” data is meaningful, the subsequent assumptions about k-space sampling (refer to point #3) are confusing and the need citations of previous literature using similar assumptions.

2. P4L115 - “If raw k-space data is available from the scanner and the acquisition sequence used to produce that data is known, then data can be re-partitioned into almost-arbitrarily small intervals.” This sentence is misleading and confusing. This sentence needs to be better explained and even pictorially illustrated. One cannot synthesize data at time points that were not acquired. Even if the data were obtained using a view sharing method, it will result in temporal blurring.

3. Simulating accelerating acquisition using parallel imaging or other methods such as compressed sensing needs to be cognizant of the fact that reduction factors do not scale directly with the total acquisition time and is dependent on the G-factor maps particular to breast images. Importantly, the phase of the undersampled synthetic data does not capture the phase artifacts in prospective acquisiton, for example.

3. Virtual scanner: The virtual scanner development is relevant to the work and aids the assumptions made of k-space trajectories and sampling intervals. However, these simulations do not take into all the effects of acquisition such as eddy currents, gradient delays, RF ringdown effects, B_1_ inhomogeneity in breast imaging to name a few. The authors are recommended to use a complete simulation software such as JEMRIS (<http://www.jemris.org/>) or integrate their virtual scanner with JEMRIS to render the simulation more comprehensive.

4. The reconstruction using a smoothness penalty per voxel and balances that with the data fidelity constraint. However, the data fidelity constraint is computed against synthetic data resulting in error propagation due to simulation, sampling and interpolation. These are noted by the authors in their comparisons with the reconstruction image quality observations being lesser compared to the inverse FFT reconstructions.

5. Most importantly, the authors claim about quantitative MRI for breast imaging in the Introduction section implies targeting of quantitative parameters for reconstruction, P1L49: “Pharmacokinetic lesion analysis requires an accurate quantitative measurement of the

50 arterial input function (AIF), which requires high temporal resolution measurements” and the subsequent analysis of different acceleration methods for improved temporal resolution. However, the measures targeted in this work are bolus arrival time and percentage enhancement time which are semi-qualitative measures for breast DCE-MRI work rather than quantitative pharmacokinetic analysis parameters of K^trans^ and v_e_. Although this is not a limitation of this study, the claims need to be clear so that the reader is aware of the capabilities and limitations of the work.

Minor comments: 

1. Abstract – L10 “and” – Italicizing is not required. The importance of both factors is implied.

2. Abstract – L11 – “conditions of breast DCE-MRI”. The word “conditions” is vague even in the abstract section

3.Abstract – L15 – “in silica” should be changed to “in silico”

4.Abstract – There is no summary of how the ECA works. There is only mention of assumptions but it is essential to relate as to how those assumptions are exploited; in one sentence.

5. P1L27 – “MRI detects cancers that other screening methods fail to detect.” This statement needs a citation to support.

6. P1L40 – “temporal resolutions of 60-90 seconds”. Please cite studies supporting this.

7. P3L86 – Challenges in coil sensitivity mapping in the case of breast MRI needs citations. Similarly, for limitations with compressed sensing. These statements indicating limitations of techniques while understood need to be referenced by citing studies that have investigated the limitations. The “learning” paragraph is poorly written which reads like a set of opinions rather than basing arguments on previously conducted studies.

8. P5L146: Table 1 is unnecessary. This could be easily summarized in a concise manner in text.

9. P11L297: “Intuitively, we can think of this optimization”. Please rephrase this to, “Intuitively, the reconstruction optimizes for ….” Phrases like “we can think” sound colloquial.

10. P18L310: “requires some amount of regularization to converge;” Please avoid vague language such as “some amount”

11. P22L396: “Voxel-wise errors in the IFFT reconstruction were generally smaller than in the ECA” Please quantify this statement with specific best and worst case scenarios – provide a range of the errors.
