# Supplementary material for: Enhancement-constrained acceleration: A robust reconstruction framework in breast DCE-MRI
Source: PLoS One. 2021 Oct 28;16(10):e0258621. doi: 10.1371/journal.pone.0258621 (PMC8553053; doi:10.1371/journal.pone.0258621)
Supplement: Attachment — Submitted filename: Response to Reviewers.docx [file pone.0258621.s007.docx]

To the PLOSone editorial team and our manuscript reviewers,

Thank you for your feedback and suggestions. In the following letter, we respond to the recommendations of our reviewers. Our responses are organized first by respondent and are further separated into major and minor comments where appropriate. Reviewer comments are formatted as block quotes in black text and our responses are directly below them in red text.

Sincerely,

Zhen Ren and Ty Easley

**PLOSone Editorial Feedback**

Please ensure that your manuscript meets PLOS ONE's style requirements, including those for file naming. The PLOSONE style templates can be found at <https://journals.plos.org/plosone/s/file?id=wjVg/PLOSOne_formatting_sample_main_body.pdf> and <https://journals.plos.org/plosone/s/file?id=ba62/PLOSOne_formatting_sample_title_authors_affiliations.pdf>

We have checked our manuscript and made adjustments to conform to PLOS ONE’s style requirements.

We note that the grant information you provided in the ‘Funding Information’ and ‘Financial Disclosure’ sections do not match. When you resubmit, please ensure that you provide the correct grant numbers for the awards you received for your study in the ‘Funding Information’ section.

| **Financial Disclosure** | **Funding Information** |
| --- | --- |
| R.F.B. was supported by the National Science Foundation via grant DMS-1654076.  <https://www.nsf.gov/>  The work described in this manuscript was supported by a grant from the National  Institutes of Health under project number 5R01CA218700-04, which was awarded to  G.S.K. <https://www.nih.gov/>  The funders had no role in study design, data collection and analysis, decision to  publish, or preparation of the manuscript. |  |

In your Data Availability statement, you have not specified where the minimal data set underlying the results described in your manuscript can be found. PLOS defines a study's minimal data set as the underlying data used to reach the conclusions drawn in the manuscript and any additional data required to replicate the reported study findings in their entirety. All PLOS journals require that the minimal data set be made fully available. For more information about our data policy, please see <http://journals.plos.org/plosone/s/data-availability>.

A public link has been added to the submission and this reviewer response, and is also now included in the GitHub repository for this paper.
<https://uchicago.app.box.com/s/3vfvdot8hzqausgr0mwfd85iwqce0dkq>

**Reviewer #1**

Major comments

In “Theory and Methods – Virtual Scanner” section: In the simulation presented in this study, path times were computed to match standard ultrafast time resolutions (i.e., sweep time = 3.5s). However, in real practice, to reach this sweep time parallel imaging would be necessary. Please address how the consideration of parallel imaging implementation would change the proposed method in the Discussion section.

We chose an idealization of standard reconstructions that we know is incorrect but produces reconstructions of optimistic quality using standard reconstruction methods. Thus, this choice of idealization puts our method at a disadvantage, giving greater confidence in any advantages our method shows. However, we also believe that ECA reconstruction is not incompatible with SENSE, and the two methods may provide complimentary information. We have updated the Discussion to reflect the reviewer’s feedback (lines 527-535 in the Tracked Changes Manuscript) with the following paragraph:

In addition, we comment on some of the assumptions made in this simulation study with respect to parallel imaging. While the current simulation assumed that the sweep time was the temporal resolution of the original images (3.5 s), in this proof-of-principle study we did not take into account that these images were acquired with parallel imaging (SENSE). However, we do not believe the ECA method to be incompatible with parallel imaging. While ECA does impose constraints on spatial data by applying masking in k-space, the reconstruction optimizes only in the temporal domain. Since SENSE spatially optimizes image data for compatibility with coil sensitivity maps, the two approaches optimize with respect to non-overlapping sources of information and thus could be combined.

In Figure 12: Please comment on the observation that ECA curves show “undershoots” at the first and last time points. Is it due to extrapolation? How is it going to affect the evaluation and kinetic analysis?

The first- and last-point undershoots do not affect the evaluation because we can easily remove those time points when calculating kinetic parameters.

The “undershoot” of the curve at the first point is a product of the fact that the center of k-space has not yet been fully sampled within the first time-window; since we initialize our solver with a zero-fill in *k*-space, this means that low-frequency components of the image are not captured in the first 250 ms. This could be solved by initializing with a static image instead of a zero-filled one. The undershoot at the last time point is effectively due to extrapolation; splines interpolate very well, but they often extrapolate poorly.

The manuscript has been updated to reflect this response.

In “Results – Bolus Arrival Time (BAT)”: lines 358 - 359 – The comments in this part are confusing. Is BAT estimated with ECA better than the one estimated with IFFT in cases 1, 3, and 4, or is BAT estimated with ECA in cases 1, 3, and 4 better than BAT estimated with ECA in cases 2 and 5? Does the substantial difference show statistical significance?

Since the simulation data is gathered from only a single trial (i.e., a single realization of the random noise), it is not possible to do significance testing. Instead, we have compiled **paired** errors in BAT arrival times at each voxel of the ECA and IFFT reconstruction; we summarize these with the median ECA error-to-IFFT error ratio between paired measurements and construct a confidence interval around each by treating each voxel error measurement as a sample from an error distribution. Aggregated across cases, the median error ratios for both lesion and vessel voxels are significantly less than 1, since 5*σ* confidence intervals for each median both have upper bounds much less than 1; from these results, we conclude that ECA estimates BAT significantly more accurately than does IFFT.

Minor Comments

In “Theory and Methods – Virtual Phantoms” section: Please provide the specific models / equations and values of associated parameters used to produce the concentration curves in vessels and lesions.

The Parker’s model is a well-known 10-parameter model to produce the concentration curves in vessels. In our AIF model, we used the same parameter setting listed in ref [13], so we did not expand the explanation of the equation and the values for each parameter. We added the equation and parameter values for the empirical model that is used to produce concentration curves in lesions on Page 7.

In “Results – Bolus Arrival Time (BAT)”: line 357 – Please correct “IFT” to “IFFT”.

The requested change has been made in the manuscript.

In Figure 10: Is the unit of colorbar percentage?

Yes, the unit of the colorbar in Figure 10 is percentage. We have added the unit in the figure.

**Reviewer #2**

Major Comments:

In silico phantom: The standard phantom for quantitiative breast DCE is the QIBA phantom (<https://qibawiki.rsna.org/index.php/Main_Page>) that provides specific qMRI pharmacokinetic parameters against which image reconstruction, image processing, regression analysis methods are typically compared. Although the idea of starting with “clinical” data is meaningful, the subsequent assumptions about k-space sampling (refer to point #3) are confusing and the need citations of previous literature using similar assumptions.

We thank the reviewer for this comment. Our experimental design prioritized the replication of patient physiology over the enforcement of a particular pharmacokinetic model. We believe that by building our phantoms from acquired data, many physiological and scanner effects (which can be very difficult to model) are essentially “baked into” the phantom, making the simulation more realistic than enforcing adherence to the two-compartment model. In addition, physiological mimicry in the phantom is important for assessing the robustness of the reconstruction: the spectral density of *k­*-space data is fully determined by physiology, and a physiologically unrealistic phantom would likely give a highly optimistic characterization of our method’s noise sensitivity.

P4L115 - “If raw k-space data is available from the scanner and the acquisition sequence used to produce that data is known, then data can be re-partitioned into almost-arbitrarily small intervals.” This sentence is misleading and confusing. This sentence needs to be better explained and even pictorially illustrated. One cannot synthesize data at time points that were not acquired. Even if the data were obtained using a view sharing method, it will result in temporal blurring.

Claiming that *k*-space data can be partitioned into arbitrarily-small intervals does not yet make any claims on the ability to produce images from that data. Rather, the claim is that *k*-space data may be partitioned into small intervals, whose length is only constrained by the temporal resolution of the scanner itself (i.e., the minimal time-separation between any two points of *k*-space data). Reconstructing an image from this data, however, is a very noise-limited process: for example, it would be extremely impractical to divide *k*-space data into intervals so small that each only contained several *k*-space points, as the SNR would be far too low for a high-quality image reconstruction. We modified the sentence to emphasize that we are re-binning *k*-space data, but a more detailed explication of the method is reserved for the Methods section.

It is also worth emphasizing that re-binning data is independent of any sampling method and is unrelated to view-sharing. Recall that a partition of a set is a division of that set into nonempty subsets such that each element is contained in exactly one subset. If we know the scan protocol and/or have timestamps for *k*-space data, then it is always possible to bin individual *k*-space data points with respect to a given partition of the acquisition time interval. A simple example case is visually illustrated in **Figure 5**.

Simulating accelerating acquisition using parallel imaging or other methods such as compressed sensing needs to be cognizant of the fact that reduction factors do not scale directly with the total acquisition time and is dependent on the G-factor maps particular to breast images. Importantly, the phase of the undersampled synthetic data does not capture the phase artifacts in prospective acquisiton, for example.

A full simulated implementation of partial-Fourier and/or parallel reconstruction was beyond the scope of this proof-of-concept study. We chose an idealization of standard reconstructions that we know is incorrect but optimistically estimates the performance of standard reconstruction methods. Thus, this choice of idealization puts our method at a disadvantage, giving greater confidence in any advantages our method shows.

Virtual scanner: The virtual scanner development is relevant to the work and aids the assumptions made of k-space trajectories and sampling intervals. However, these simulations do not take into all the effects of acquisition such as eddy currents, gradient delays, RF ringdown effects, B_1_ inhomogeneity in breast imaging to name a few. The authors are recommended to use a complete simulation software such as JEMRIS (<http://www.jemris.org/>) or integrate their virtual scanner with JEMRIS to render the simulation more comprehensive.

The purpose of our study is to generate new k-space trajectories and to validate the ECA method using a small portion of k-space based on these new sampling trajectories. For this particular purpose, we chose to build up our own simulation scanner which provides flexibility to generate the desired k-space trajectories. In later work, we will consider a more complex simulation model that includes motion artifacts, T2* effect and other major effects of acquisition.

The reconstruction using a smoothness penalty per voxel and balances that with the data fidelity constraint. However, the data fidelity constraint is computed against synthetic data resulting in error propagation due to simulation, sampling and interpolation. These are noted by the authors in their comparisons with the reconstruction image quality observations being lesser compared to the inverse FFT reconstructions.

As the reviewer acknowledges, the image quality is lower in the ECA reconstruction than in the IFFT reconstruction, though voxel intensity errors are very small (<1%) in both reconstructions. This is a trade-off inherent to the optimization underlying the image reconstruction, not a feature of the simulated data. That said, the models behind the simulation could certainly benefit from (e.g.) more sophisticated noise-modeling. The noise and signal model properties are of course relevant to the performance of the reconstruction algorithm, and (as we note in the manuscript) we hope to further characterize their influence in future research.

Most importantly, the authors claim about quantitative MRI for breast imaging in the Introduction section implies targeting of quantitative parameters for reconstruction, P1L49: “Pharmacokinetic lesion analysis requires an accurate quantitative measurement of the 50 arterial input function (AIF), which requires high temporal resolution measurements” and the subsequent analysis of different acceleration methods for improved temporal resolution. However, the measures targeted in this work are bolus arrival time and percentage enhancement time which are semi-qualitative measures for breast DCE-MRI work rather than quantitative pharmacokinetic analysis parameters of K^trans^ and v_e_. Although this is not a limitation of this study, the claims need to be clear so that the reader is aware of the capabilities and limitations of the work.

We thank the reviewer for their comment and have edited the work to more accurately specify the scope of our claims; in particular, we emphasize that we focus on capturing the bolus arrival time in a physiologically realistic phantom, which is a necessary prerequisite for quantitative pharmacokinetic analysis.

Minor comments: 

Abstract – L10 “and” – Italicizing is not required. The importance of both factors is implied.

The requested change has been made in the manuscript.

Abstract – L11 – “conditions of breast DCE-MRI”. The word “conditions” is vague even in the abstract section

The requested change has been made in the manuscript.

Abstract – L15 – “in silica” should be changed to “in silico”

The requested change has been made in the manuscript.

Abstract – There is no summary of how the ECA works. There is only mention of assumptions but it is essential to relate as to how those assumptions are exploited; in one sentence.

The requested change has been made in the manuscript.

P1L27 – “MRI detects cancers that other screening methods fail to detect.” This statement needs a citation to support.

The requested change has been made in the manuscript.

P1L40 – “temporal resolutions of 60-90 seconds”. Please cite studies supporting this.

The requested citation(s) have been added.

P3L86 – Challenges in coil sensitivity mapping in the case of breast MRI needs citations. Similarly, for limitations with compressed sensing. These statements indicating limitations of techniques while understood need to be referenced by citing studies that have investigated the limitations.

The “learning” paragraph is poorly written and reads like a set of opinions, rather than basing arguments on previously conducted studies.

The requested citation(s) have been added, and the learning paragraph has been cut from the manuscript.

P5L146: Table 1 is unnecessary. This could be easily summarized in a concise manner in text.

The requested change has been made in the manuscript.

P11L297: “Intuitively, we can think of this optimization”. Please rephrase this to, “Intuitively, the reconstruction optimizes for ….” Phrases like “we can think” sound colloquial.

The requested change has been made in the manuscript.

P18L310: “requires some amount of regularization to converge;” Please avoid vague language such as “some amount”

The requested change has been made in the manuscript.

P22L396: “Voxel-wise errors in the IFFT reconstruction were generally smaller than in the ECA” Please quantify this statement with specific best and worst case scenarios – provide a range of the errors.

We have provided the numbers in Table 4.
